# Case Report: A case of craniopharyngioma in the cerebellopontine angle

**DOI:** 10.3389/fonc.2026.1786109

**Published:** 2026-03-05

**Authors:** Mulin Zhang, Chengyue Zhu, Yuping Diao, Yin Mo

**Affiliations:** Department of Medical Imaging, The First Affiliated Hospital of Kunming Medical University, Kunming, Yunnan, China

**Keywords:** adamantinomatous craniopharyngiomas, cerebellopontine angle region, computed tomography, craniopharyngioma, magnetic resonance imaging

## Abstract

We report a rare case of craniopharyngioma located in the cerebellopontine angle. A 36-year-old male patient presented with relevant clinical symptoms. Computed tomography (CT) revealed a slightly hypodense mass in the left cerebellopontine angle extending to the ambient cistern. Magnetic resonance imaging (MRI) demonstrated an irregular lesion in the same region, which was slightly hyperintense to isointense on T1-weighted images and heterogeneously hyperintense on T2-weighted images. Diffusion-weighted imaging (DWI) showed mixed signals. The lesion was surgically resected, and the final pathological diagnosis confirmed it as a craniopharyngioma.

## Introduction

A 36-year-old man presented with a two-year history of headaches. His symptoms significantly progressed in January 2023, when he developed sudden-onset, unexplained hearing loss in the left ear, accompanied by dizziness, headache exacerbation, and left-sided facial numbness persisting for several months. On February 23, 2023, an MRI performed at a local hospital revealed an occupying lesion in the left cerebellopontine angle (CPA) region. Consequently, he was admitted to our hospital for further evaluation and management on March 7, 2023.Upon admission, physical examination indicated that the patient was in fair general condition and was fully conscious. Visual acuity and visual fields were normal in both eyes. A marked decrease in hearing was noted in the left ear, while hearing in the right ear was approximately normal. Cranial nerve examination revealed left-sided facial hypoesthesia. The Romberg test was positive, but the finger-to-nose test was performed steadily and accurately. Both deep and superficial sensations were symmetrically intact throughout the body. All physiological reflexes were present, with no pathological reflexes elicited. Examination of the autonomic nervous system was unremarkable. Pituitary hormone profiling demonstrated a low level of adrenocorticotropic hormone (ACTH). Subsequently, cranial CT and MRI examinations were completed at our institution for comprehensive preoperative assessment.

## Imaging findings

**Figure 1:**
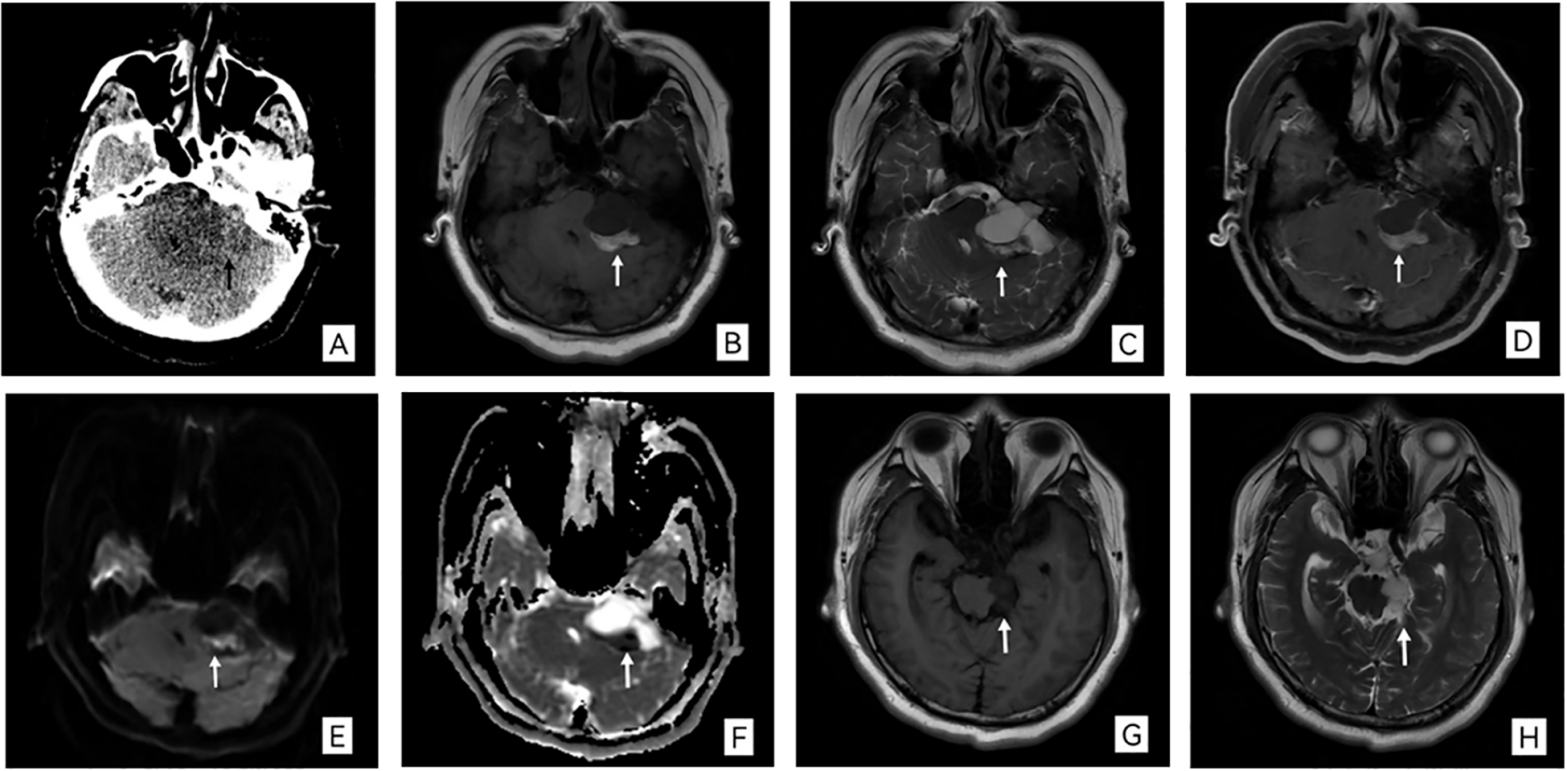
**(A)** Axial non-contrast CT image demonstrates a hypodense mass (black arrow) in the left cerebellopontine angle and ambient cistern, exerting mass effect on the adjacent parenchyma. **(B, C)** Axial T1- and T2-weighted MR images show the corresponding irregular lesion (white arrow). **(D)** Axial contrast-enhanced T1-weighted MR image reveals prominent enhancement of the tumor margins and internal septations, while the cystic components remain non-enhancing. **(E, F)** Diffusion-weighted imaging (DWI) and the corresponding apparent diffusion coefficient (ADC) map display heterogeneous signal within the mass. **(G, H)** Sagittal and coronal T1-weighted MR images, respectively, illustrate the lesion’s extent and its relation to the suprasellar cistern.

(1) Cranial CT: Non-contrast CT revealed a well-defined, slightly hypodense mass in the left cerebellopontine angle region, extending into the ambient cistern. The lesion caused significant mass effect, compressing the brainstem and fourth ventricle, resulting in obstructive hydrocephalus with dilation of the supratentorial ventricles (**Figure 1A**). No appreciable enhancement was observed following contrast administration.

(2) Cranial MRI: MRI confirmed an irregular, lobulated mass in the left cerebellopontine angle. The lesion demonstrated heterogeneous signal intensity: it was predominantly isointense to brain parenchyma with focal hyperintense areas on T1-weighted images, and heterogeneously hyperintense on T2-weighted images. Diffusion-weighted imaging (DWI) showed a mixed signal pattern. Notably, the mass extended into and slightly widened the ipsilateral internal acoustic meatus. Post-contrast T1-weighted images showed prominent enhancement of the lesion’s peripheral margins and internal septations. The lesion measured approximately 4.3 cm×3.5 cm×4.0 cm (**Figures 1B, C**).

## Preliminary radiological diagnosis

Based on the location (CPA with internal auditory canal extension), imaging characteristics, and enhancement pattern, the initial diagnosis favored an acoustic neuroma (vestibular schwannoma), with the possibility of intratumoral hemorrhage or cystic degeneration.

## Surgery

The patient underwent resection of the left posterior fossa lesion under general anesthesia on March 14, 2023. A left retrosigmoid approach was utilized. A curvilinear incision was made behind the left ear along the hairline. A suboccipital craniotomy (approximately 7 cm × 6 cm) was performed, exposing the transverse-sigmoid junction. The dura mater was opened in a cruciate fashion. Cerebrospinal fluid was released from the cisterna magna to facilitate brain relaxation. Upon gentle retraction of the left cerebellar hemisphere, a tumor appeared as grayish-yellow tissue with a moderately firm and slightly tenacious consistency. It was well-demarcated from the adjacent brain parenchyma. The left facial and acoustic nerves were compressed and displayed an abnormal morphology. Microsurgical resection was performed with the aid of intraoperative neurophysiological monitoring. The tumor was debulked internally using an ultrasonic aspirator, followed by careful dissection of the tumor capsule from the surrounding neurovascular structures. A gross total resection was achieved. After hemostasis, meticulously suture and repair the dura mater using an artificial dura patch. Place one epidural drain. Replace the cranial bone flap and repair the bone defect with synthetic material. Perform layered closure of the muscle, subcutaneous tissue, and skin layers. Postoperatively, the patient recovered uneventfully. The facial numbness and hearing impairment on the left side were significantly alleviated following the tumor resection. However, the patient exhibited mild facial paralysis, manifesting as incomplete closure of the left eyelid 10 days after the operation. No other specific complaints were noted. He was discharged with instructions to undergo adjuvant chemoradiotherapy.

## Immunohistochemistry findings

Pathological results of intraoperative samples showed that β-catenin, VIM, and CD34(vascular structures, as a normal control) are positive. The tumor cells were negative for S-100, CD56, MBP, CD57, EMA, KI-67, Pan-CK, SOX-10, and GFAP (β-catenin: beta-catenin; VIM: vimentin; CD34: hematopoietic progenitor cell antigen CD34; S-100: S100 calcium-binding protein; CD56: neural cell adhesion molecule (NCAM); MBP: myelin basic protein; CD57: HNK-1/human natural killer-1; EMA: epithelial membrane antigen; KI-67: marker of proliferation Ki-67; Pan-CK: pan-cytokeratin; SOX-10: SRY-box transcription factor 10; GFAP: glial fibrillary acidic protein).

**Figure 2:**
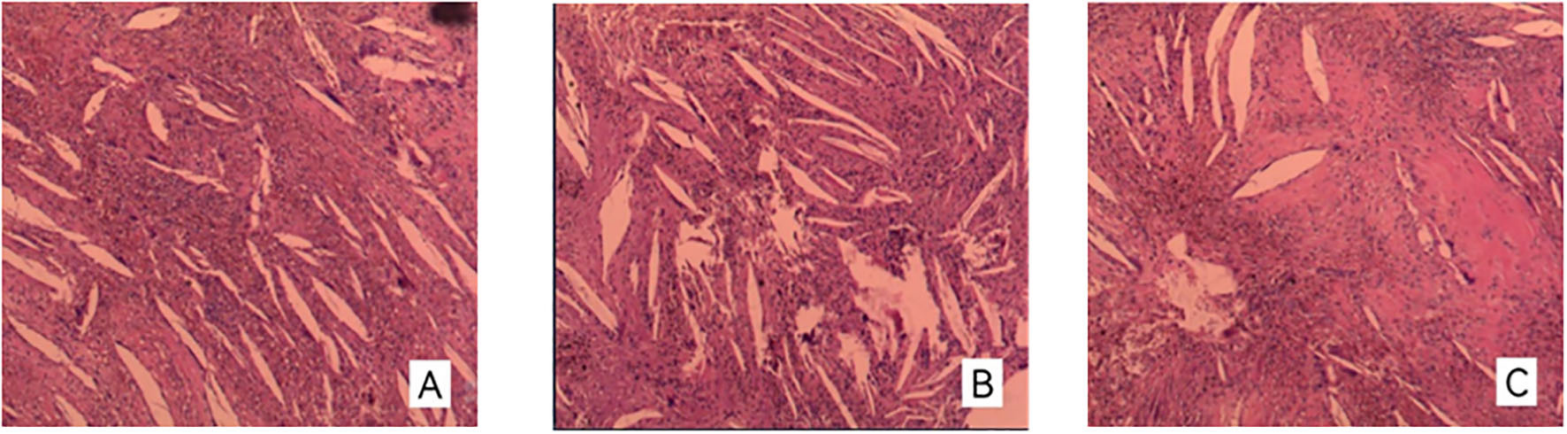
**(A–C)** Pathological findings reveal a craniopharyngioma with extensive cholesterol crystals.

The initial postoperative pathology at our institution described the lesion as a calcifying epithelioma with granulomatous inflammation. Subsequently, the specimens were referred for consultation to the Department of Pathology at Huashan Hospital, Fudan University. The expert review established the final diagnosis as craniopharyngioma, adamantinomatous type (ACP), with notable features of extensive cholesterol cleft formation (**Figure 2**).

## Discussion

Craniopharyngioma is classified as a WHO Grade I tumor, typically arising from the proliferation of residual epithelial cells in the Rathke’s pouch or from squamous metaplasia of epithelial cells. Craniopharyngiomas exhibit two peak incidence periods: the first between ages 5 and 15, and the second around age 50 (1). Most tumors arise suprasellar and intrasellar (2). They grow slowly, typically presenting clinical symptoms only when the tumor is large. These symptoms include visual impairment, increased intracranial pressure, diabetes insipidus, and headaches caused by compression of structures such as the optic chiasm, ventricular system, pituitary gland, and hypothalamus. These conditions can severely impact long-term survival and quality of life (3).Pathologically, craniopharyngiomas are classified into two types: Adamantinomatous craniopharyngioma (ACP) and Papillary craniopharyngioma (PCP) (4). ACP predominantly affects children aged 5–15 and adults aged 45–60, while PCP generally occurs only in adults (5, 6).The pathogenesis of these two craniopharyngioma subtypes also differs. ACP is believed to arise from mutations in the CTNNB1 gene within epithelial cells retained from Rathke’s pouch during embryogenesis, activating the WNT pathway and leading to tumor formation. PCP, conversely, is thought to result from BRAF V600E gene mutations that activate the MAPK signaling pathway, causing squamous metaplasia and subsequent tumorigenesis (7). The imaging manifestations of craniopharyngioma can be categorized into cystic, cystic-solid, and solid types, with solid forms being less common and cystic and cystic-solid types more prevalent. On computed tomography (CT), craniopharyngiomas typically present as well-demarcated lesions. The cystic components are characteristically hypodense, whereas the solid portions may appear isodense, heterogeneous, or slightly hyperdense relative to brain parenchyma. Calcifications are a common feature, often observed within the solid nodules and along the cyst walls.

On MRI, cystic components usually demonstrate low signal intensity on T1-weighted images (T1WI), which may increase to high signal if the fluid contains abundant cholesterol crystals or high protein content. On T2-weighted images (T2WI), these areas typically show high signal intensity, which can be heterogeneous. Solid components typically appear isointense or of heterogeneous signal on T1WI and exhibit high signal intensity on T2WI. Following contrast administration, both the solid components and the cyst walls usually demonstrate marked enhancement. Craniopharyngiomas in this location are relatively rare. The symptoms exhibited in this case—hearing loss, vertigo, and headache—result from tumor compression of adjacent nerves and brain tissue. Clinically, these symptoms lack diagnostic specificity. The patient’s age at onset aligns with the second peak incidence period for craniopharyngiomas, yet the imaging findings and location differ from those of typical craniopharyngiomas. Based on existing literature theories, craniopharyngioma occurring in the CPA regionis speculated that this may result from craniopharyngioma cells migrating to the posterior fossa after cerebellar formation, possibly via cerebrospinal fluid flow. Some recurrence cases may stem from migration along surgical tracts to other sites (8), though the exact cause remains unclear. Neurons in the paraventricular nucleus (PVN) of the hypothalamus have clear axonal collateral projections to the craniopharyngioma region. Several types of sellar region tumors, including craniopharyngiomas (such as pituitary adenomas and germ cell tumors), may promote their own growth by hijacking the axonal collateral projections of hypothalamic neurons. Similar cases include a occurring in a patient with familial adenomatous polyposis (9), and a patient presenting with garden syndrome (10). Imaging differentiation is required from other cerebellopontine angle (CPA) lesions, including acoustic neuromas, tuberculum sellae meningiomas, cavernous hemangiomas, and intracranial epidermoid cysts (**Table 1**). Acoustic neuroma (vestibular schwannoma) typically presents as an enhancing mass centered on the internal auditory canal. On T1-weighted imaging (T1WI), it usually exhibits isointense to slightly hypointense signal relative to brain parenchyma. Post-contrast scans demonstrate homogeneous or heterogeneous enhancement; cystic components, if present, may show peripheral rim enhancement. Tuberculum sellae meningioma predominantly affects middle-aged women, non-contrast CT often shows a hyperdense mass, with homogeneous intense enhancement post-contrast, on MRI, characteristic features include a broad dural base, homogeneous enhancement, and the “dural tail sign”. Cavernous hemangioma (cavernoma) has a distinctive “popcorn” appearance on MRI due to mixed signal intensities from blood products of varying ages, it is typically surrounded by a complete hypointense rim of hemosiderin on T2-weighted images and exhibits marked hypointensity (“blooming”) on susceptibility-weighted imaging (SWI), with little to no contrast enhancement. Intracranial epidermoid cyst, also known as a cholesteatoma, this lesion exhibits insinuating (“creeping”) growth along spaces. It rarely shows cyst wall calcification, on diffusion-weighted imaging (DWI), it characteristically shows restricted diffusion (high signal), which is a key distinguishing feature. Most epidermoid cysts do not enhance; mild peripheral enhancement may be seen in a minority of cases.

**Table 1:** Differential diagnosis of CPA region tumors.

Lesion type	Gender/Common age of onset	Common location	MRI characteristics (Non-contrast)	MRI characteristics (Contrast-enhanced)
Acoustic Neuroma/Vestibular Schwannoma	No gender different	Early: internal auditory canal (IAC) Late: CPA (cerebellopontine angle) to IAC	Shape: funnel-shaped (IAC expansion). Signal: T1 iso/hypointense, T2 iso/hyperintense. Texture: solid, cystic, or mixed.	“Ice-cream cone” sign: solid part shows intense enhancement; cystic part does not enhance.
	Age: 30-50			
Tuberculum Sellae Meningioma/Sellar Region Meningioma	Female> Male	Centered on tuberculum sellae; midline or paramidline.	Shape: round/semi-circular (sessile). Signal: T1 iso/hypointense, T2 slightly hyperintense. borders: clear borders; the optic chiasm is pushed upwards, may encase optic nerve.	Homogeneous & intense enhancement. “Dural tail” sign; typically encases/displaces ACA or ICA.
	Age:30-40 (Adults)			
Cavernous Hemangioma	Female> Male	Intra-axial: temporal lobe. extra-axial: parasellar/cavernous sinus.	Intracerebral type: exhibits a characteristic ‘popcorn’ appearance with a mixed-signal core and a hypointense hemosiderin rim. Extracerebral type: presents as well-defined, dumbbell-shaped lesions lacking flow voids, calcification, or significant internal vascularity.	Enhancement varies based on content.
	Age: 40-50			
Intracranial Epidermoid Cyst	No gender different	CPA, basal cisterns, parasellar, intraventricular.	Shape: irregular, “cauliflower-like” Growth: “creeping/Insinuating” growth around nerves/vessels. Signal: similar to CSF (T1 low, T2 high). Key feature: DWI hyperintense (restricted diffusion).	No enhancement of the substance. Rarely shows rim enhancement.
	Peak: ~40			

## Data Availability

The raw data supporting the conclusions of this article will be made available by the authors, without undue reservation.
